# Improved ABC Algorithm Optimizing the Bridge Sensor Placement

**DOI:** 10.3390/s18072240

**Published:** 2018-07-11

**Authors:** Jianhui Yang, Zhenrui Peng

**Affiliations:** 1 School of Electronic and Information Engineering, Lanzhou Jiaotong University, Lanzhou 730070, China; yangjh@mail.lzjtu.cn; 2 School of Mechatronic Engineering, Lanzhou Jiaotong University, Lanzhou 730070, China

**Keywords:** sensor placement, artificial bee colony algorithm, dynamic random coverage coding, matching & preserving strategy, Ha-Qi long span railway bridge

## Abstract

Inspired by sensor coverage density and matching & preserving strategy, this paper proposes an Improved Artificial Bee Colony (IABC) algorithm which is designed to optimize bridge sensor placement. We use dynamic random coverage coding method to initialize colony to ensure the diversity and effectiveness. In addition, we randomly select the factors with lower trust value to search and evolve after food source being matched in order that the relatively high trust point factor is retained in the exploitation of food sources, which reduces the blindness of searching and improves the efficiency of convergence and the accuracy of the algorithm. According to the analysis of the modal data of the Ha-Qi long span railway bridge, the results show that IABC algorithm has faster convergence rate and better global search ability when solving the optimal placement problem of bridge sensor. The final analysis results also indicate that the IABC’s solution accuracy is 76.45% higher than that of the ABC algorithm, and the solution stability is improved by 86.23%. The final sensor placement mostly covers the sensitive monitoring points of the bridge structure and, in this way, the IABC algorithm is suitable for solving the optimal placement problem of large bridge and other structures.

## 1. Introduction

Civil engineering structures, such as large span bridges and super high-rise buildings, are developing towards a direction of oversize and complexity [1,2]. In the face of these complex structural characteristics, as well as external natural disasters and artificial weight load, it is very important to carry out intelligent monitoring of civil engineering. Sensors are to be installed at key parts of the structure to obtain internal physics and force characteristics data for bridges as well as other civil structures data, then perform scientific numerical calculations to assess the degree of structural damage and safety indicators. This is of great practical significance for the daily maintenance of structures, improving the safety and durability of structures, ensuring the safety of people’s lives and property, and making the structure better serve the economic construction [3,4,5].

Sensor optimization placement is a key part of the entire intelligent monitoring work. A good bridge sensor placement is based on the following principles: (1) In situations where all types of noise are objectively present, the fewer sensors installed in the right place, the better the information obtained for more structural response data; (2) The measured structural response data can reverse the theoretical numerical analysis results, etc. [6,7]. In theory, the greater the number of sensors deployed, the more accurate the resulting response data that can be obtained. However, while this is still feasible for small bridges and other structures, for large and complex bridges, the corresponding hardware facilities, transmission, storage and other costs will be greatly increased, which is not conducive to the actual implementation of the project [8,9].

The optimal placement of sensors in the bridge structure mainly involves the solution of the number and location of sensors, which is a typical Non-deterministic Polynomial Hard (NP-Hard) problem [10,11]. The difficulty of solving this problem is greatly affected by the actual data dimension. How to solve the problem efficiently and accurately has become one of the research hotspots in this field. Many scholars have conducted extensive research on the issue of the optimization of sensor placement. Kammer [12] proposed a well-known effective independent method for the optimization of sensor placement for large space structures in 1991; Heo [13] used the maximum structure motion energy obtained from the test point as the sensor optimization target and proposed a sensor placement method based on the motion energy method. The modal strain energy MSE and the modality guarantee criterion MAC are regarded as the fitness function and the improved genetic algorithm is used to study the optimization problem of spatial structure sensors by Liu et al. [14]. Peng [15] introduced the chaos search strategy and binary coding into the monkey algorithm to solve the problem of optimal placement of suspension bridge sensors. The issue of solving the complete structural performance evaluation of high-rise buildings with multiple types of sensor placement and response reconstruction methods was proposed by Rong et al. [16]. And there are many other sensors optimization and application problems in bridge.

On the other hand, inspired by the behavioral processes of animals, insects and other groups, the swarm intelligence algorithms appeared in 1980s and has attracted wide attention from many scholars. Such representative algorithms mainly include Genetic Algorithm (GA) [17], Ant Colony (ACO) algorithm [18], Particle Swarm Optimization (PSO) algorithm [19], Artificial Fish School (AFSA) algorithm [20], Monkey algorithm (MA) [21], Artificial Bee Colony (ABC) algorithm [22], etc. In recent years, a large number of scholars have been keen to use this group of intelligent algorithms to solve the problem of optimal placement of sensors in various fields. Yan et al. [23] used genetic algorithms to solve the problem of fixed-point sensor location optimization on expressways. Rao et al. [24] have improved the particle swarm algorithm to make it suitable for the sensor optimization placement problem and applied it to the sensor optimization placement of a large spatial structure, which had achieved good results. Yi et al. [25] proposed an adaptive monkey algorithm and adopted a dual-structure encoding and automatic climbing strategy to increase the global search ability and solve the problem of high-rise building sensor placement optimization. Peng et al. [26] used artificial fish swarm algorithm to solve the problem of optimal sensor placement and applied the behaviors of four typical artificial fish groups to search for the optimal solution. Compared with the particle swarm optimization algorithm, the authors obtained a good sensor placement result. This type of swarm intelligence algorithm is applied to solve the problem of sensor optimization placement, one solves a specific type of problem and another improves the convergence rate of the algorithm or increases the accuracy of the algorithm. They all show different degrees of advantages [27,28,29].

Inspired by the bee-gathering process, Karaboga [30] first proposed a new intelligent algorithm named Artificial Bee Colony (ABC) algorithm in 2005. The algorithm has the advantages of a simple principle, few control parameters, etc. It is widely used in function optimization, neural network training, production scheduling and other engineering fields [31,32]. Li et al. [33] studied the optimization of numerical functions using artificial bee colony algorithm. ABC algorithm was used to optimize the training of a neural network with two hidden layers by Adak et al. [34]. Yasin et al. [35] used the improved ABC algorithm in power system harmonic estimation applications and obtained good estimation results. Fereydoun et al. [36] proposed the ABC-CA urban growth model to simulate the urban growth process of Urmia (Iran). Of course, there are many other applications of artificial bee colony algorithms.

However, the basic ABC algorithm has the defects of being easily trapped in local optima and other problems. For this reason, some scholars have also put forward many improvement strategies. Alatas [37] used chaotic mapping theory to initialize the ABC algorithm, in order to increase individual diversity and prevent the algorithm from falling into local optimization. Gao [38] introduced differential evolution and selection probability, improved the artificial bee colony algorithm by using global optimization information, and made the algorithm perform better in solving complex numerical optimization problems. Liu et al. [39] used the Logistic equation to generate the initial population in the artificial bee colony algorithm and improved the search strategy in the detection bee stage, which have improved the algorithm’s convergence rate and optimization ability. Akay [40] used the globally optimal individuals to guide the search direction of the bees in order to increase the search ability of the ABC algorithm. Gao et al. [41] improved the search performance of the ABC algorithm by adaptively adjusting the search range and calculating the transition probability. Zhu et al. [42] were inspired by the PSO algorithm idea and added the heuristic formula of the current optimal individual to the standard ABC algorithm, making the search of the algorithm more prone. Jiang et al. [43] proposed a weighted global artificial bee colony algorithm, which used the global optimal solution information to promote food source evolution, improved the convergence rate of the algorithm, and was applied in the optimization deployment of gas sensors.

When solving specific engineering problems, especially in solving combinatorial optimization problems such as optimal placement of sensors, the initialization of individuals in the ABC algorithm and the evolution strategy of the bee-hogging honey will directly affect the optimal solution search speed and accuracy of the sensor placement scheme. Considering that different sensor placement numbers correspond to different coverage densities, differential evolutionary thinking and multiple food source matching factor trust values have different characteristics [44], based on the ABC algorithm, this paper presents an improved artificial bee colony algorithm named IABC for optimal placement of bridge sensors. By dynamically combining the sensor placement density, the initialization method of the basic ABC algorithm is improved through Dynamic Random Coverage Coding (named DRCC) initialization method, and the diversity of food source initialization is increased. According to the characteristics of diversity and selectivity of the elements after multiple food sources matching, the evolutionary mechanism of Matching & Preserving Strategy (named MPS) is proposed to optimize bee colony evolutionary strategies and to improve the convergence rate and the global search efficiency of the algorithm. Further, the paper verifies the feasibility and effectiveness of the algorithm through a bridge example.

The remainder outline of this paper is as follows: Section 2 explains the mathematical model of the optimal placement of the bridge sensor and the determination of the objective function. Section 3 introduces the basic principle and steps of ABC algorithm. A detailed description of the IABC algorithm proposed in this paper combining the practical problems in the optimal placement of bridge sensors is given in Section 4. Section 5 shows the feasibility and effectiveness of the IABC algorithm by a bridge example and gives the final solution results. Finally, Section 6 summarizes the analysis results of the improved algorithm and the examples.

## 2. Mathematical Model of Bridge Sensor Optimal Placement

Damage diagnosis of bridge structures is a dynamic detection technology developed in recent years. The commonly accepted method of damage assessment is experimental modal analysis at home and abroad [45,46]. The first step of modal test is to identify the location of the bridge structure response test points. However, large-scale bridge structures have more measurable degrees of freedom, and it is not practical to obtain response information in all degrees of freedom. Therefore, it is necessary to optimize the position of the limited measuring points.

According to the principle of structural dynamics, the modal vectors build a set of orthogonal ones. In practical engineering, because of the limitation of measurable freedom, it is hard to get a complete modal vector. At the same time, due to the influence of noise, the actual incomplete modal vectors do not satisfy the orthogonal conditions, even in extreme cases, the orthogonality will be lost because the space angle between the modal vectors is too small. Therefore, when selecting the measuring points, it is necessary to keep the measured modal vectors at a large spatial intersection angle, so as to maximize the structural dynamic characteristics as much as possible [47,48,49].

Carne et al. [50] consider that the modal confidence matrix (MAC matrix) is an effective tool to evaluate the intersection angle of modal vectors. The MAC matrix can be expressed as:

(1) $$MAC_{ij}=\frac{{\left(\varphi_{i}^{T}\varphi_{j}\right)}^{2}}{\left(\varphi_{i}^{T}\varphi_{i}\right)\left(\varphi_{j}^{T}\varphi_{j}\right)},$$

where $\varphi_{i}$ and $\varphi_{j}$ respectively represent the ith and jth mode shape vectors. The non-diagonal element $MAC_{ij}\left(i\neq j\right)$ can reflect the intersection angle between the two mode shape vectors. The smaller the value, the better the independence of the test freedom of each order, and the better the sensor placement is. If it is another case, the worse the result [51]. As a result, the placement of the measurement points should strive to minimize the maximum non-diagonal element of the corresponding MAC matrix.

In the Finite Element Analysis (FEA) of a bridge structure, the number of nodes depend on the structure model and the nodes number in its turn determine the precision of finite element model. Each node of the FEA has 6 Degrees of Freedom (DOF) which are translation Ux,Uy,Uz and rotation *R*x,Ry,Rz. From them one DOF (it may be named UxDOF, etc.) can be selected according to the highest participatory quality results of mode shape and its modal vectors are used to form the mode shape matrix $\emptyset_{D\times L}$ as the basic research data. Where D is the number of nodes in the FEA model, L is a selected modal order. In all nodes, m nodes are selected as sensor placement points. That is, selecting m rows data in $\emptyset_{D\times L}$ constitute a MAC matrix so that its maximum non-diagonal element tends to be minimum, and relatively good sensor placement results can be achieved.

Based on the modal vector relationship of the MAC matrix and the demand for bridge sensor placement, the objective function fit is constructed as follows:

(2) $$\;fit\left(x\right)=max\left\{MAC_{ij}\right\},\left(i,j\in \left[1,L\right],i\neq j\right),$$

where x is node number of the sensors position and also the solution vector of the problem. The smaller the objective function value fit(x), the better the independence of the test freedom, and the better placement of the sensors for the test node x. Aiming at the problem of bridge sensor optimal placement and bridge mode data, therefore, the goal of this paper is selecting the appropriate node number x to make the objective function fit(x) as minimal as possible.

For a large bridge structure with several hundred meters even has more than one thousand nodes. Obviously, it is difficult to solve the optimal value of the objective function by enumeration. The swarm intelligence algorithm has the advantages of fast speed and high precision in solving multi-dimensional optimization problems and is widely applied to various engineering examples. ABC algorithm is a relatively good swarm intelligence algorithm, which can be tried to solve the problem of the bridge sensor optimal placement.

## 3. The Basic ABC Algorithm

The basic ABC algorithm consists of three kinds of bees which are employed bees, onlooker bees, and scout bee [32]. The number of employed bees and onlooker bees are the same as that of food sources. Each food source is a potential solution to the optimization problem. The pros and cons of food sources are generally represented by “adaptation values” which reflect the quality of the corresponding solutions. The steps of the basic ABC algorithm can be described as follows:

Step 1: Set the parameter of the algorithm and initialize the food source. Assuming that the number of food sources is SN, every individual is a D dimension data, then the ith (i=1, 2, 3,⋯,SN)  food source can be expressed as:

(3) $$\;X_{i}=\left\{x_{i1},x_{i2},\cdots ,x_{iD}\right\}.$$

Step 2: Calculate the objective function value and fitness value corresponding to each food source. In some cases, the fitness value may not be calculated, and the quality of the solution may be directly evaluated by the objective function value. This paper uses the objective function as the quality standard for the evaluating solution. The smaller the objective function value, the higher the quality of the corresponding solution.

Step 3: The employed bee conducts a random search of the attached food source according to Equation (4):

(4) $$\;V_{i}=\left\{x_{i1},x_{i2},\cdots ,x_{ipara}+rand\left(-1,1\right)\left(x_{ipara}-x_{kpara}\right),\cdots ,x_{iD}\right\},$$

where $V_{i}$ represents a new source conducted by employed bee based on the food sources $x_{i}$, para is a random integer between 1~D and rand(−1,1) is a random number within the range of [−1,1], $x_{k}$ is a random selection of the neighbor food sources, $x_{ipara}$ and $x_{kpara}$ are the $para^{th}$ dimension values of their corresponding food sources respectively (para cannot be transgression). And then, according to the greed rules, decide whether to retain the new food source $V_{i}$. If the fitness value of $V_{i}$ is better than $x_{i}$, then $x_{i}$ is discarded, and the number of stagnation of $V_{i}$ is set to 0. In the contrary case, $V_{i}$ is better to be discarded and the number of stagnation of $x_{i}$ is added one.

Step 4: Calculate the selection probabilities of all food sources. The probability of selection for each food source is calculated as in Equation (5):

(5) $$\;P_{i}=\frac{fit_{i}}{\sum_{j=1}^{SN}fit_{j}}$$

where $fit_{i}$ and $fit_{j}$ are the fitness values of the corresponding food sources. The greater the food source’s fitness value is, the greater the probability that the food source will be selected.

Step 5: Onlooker bee exploits the food source for secondary recovery according to Equation (5). When the onlooker bee selects a food source, it searches the new food source according to Formula (4) just like the employed bee. Then the new food source is determined according to the greed rule, and the number of stagnation is marked correspondingly. In this process, food sources with higher quality may be exploited many times, resulting in a significant increase in the overall quality of the group.

Step 6: When the maximum stagnation frequency of a food source exceeds limit in all food sources, the food source is discarded and a new food source is randomly generated in the global scope. Since there is only one Scout bee, only the food source with the largest number of stalls is processed at a time.

Step 7: Determine whether the algorithm satisfies the termination condition. If so, the algorithm terminates; otherwise, the process continues to step 3 until the iteration is completed.

**Figure 1:**
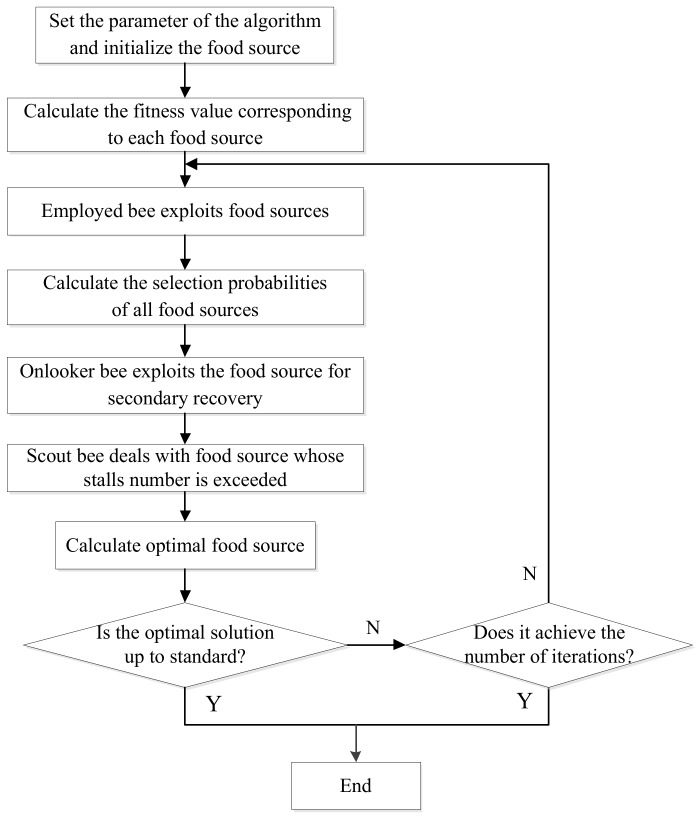
The flowchart of the basic ABC algorithm.

The flowchart of the basic ABC algorithm is shown in Figure 1.

The basic ABC algorithm has many advantages such as fast convergence rate and few input parameters, but there are still some problems in solving different specific problems. For example, in the process of resolving the problem of combinatorial optimization of bridge sensors, the ABC algorithm is easily trapped into local optimum and has low search capability. Aiming at this problem, combined with the actual needs of bridge sensor placement, we propose an initialization mechanism of DRCC and an evolutionary method of MPS, in order to improve the application of basic ABC algorithm in sensor optimal placement.

## 4. Improved ABC(IABC) Algorithm

When the basic ABC algorithm is used to solve the problem of bridge sensor placement, we use binary coding to express the solution, in other words, the binary coding of every food source corresponds to a feasible solution of the sensor placement. A “0” solution space element means no sensor installed in the corresponding node, otherwise, a sensor is placed. The individual food source is usually divided into 0–1 parts by means of random number threshold. This method is simple and easy to implement, but for bridges with large DOF, the initialization results are difficult to be limited by the number of bridge sensors. In addition, due to probabilistic factors, the initialization of the coding results tends to concentrate in the local dimension, and the individual diversity cannot be guaranteed. It is considered that the ratio of the bridge sensor number to the total number of bridge structural freedom dynamically changes, and the information reflects the density of the sensor placement. However, in the initialization process, the value in which dimension is determined to be “1” is an embodiment of the sensor placement density and dispersion. With reference to the coverage density information of the sensor placement, an initialization method for DRCC is proposed.

On the other hand, when the bee colony in the basic ABC algorithm searches and updates the food source, if only the neighboring food source is considered to have 0–1 inversion on the corresponding dimension, the reference information is slightly little. The evolutionary approach has a certain degree of blindness, resulting in poor optimization speed and accuracy. In addition, this mode of operation will change the number of 0 & 1 in the individual food source, which is not conducive to the search for better value. Liu et al. [52] introduced the differential evolution crossover operation into the evolution of ABC algorithm. The application of multidimensional neighborhood search strategy improved the convergence rate of ABC algorithm and solved the problem of 0–1 knapsack. Inspired by the difference and selectivity of the food source matching factors and literature [52,53,54], this paper proposes a food source evolution mechanism with MPS. Through transverse comparison with the neighborhood food sources, we can identify the factor information with high trust value and make a selective evolution of factor segments with low trust value. This method can make the potential solutions of food sources better on the basis of excellent factors.

### 4.1. Initialization of DRCC

Suppose that D nodes are used to establish FEA model for a certain bridge structure, and m nodes are selected as the points where to place the sensors. When initializing SN food sources whose elements are binary value set, if a function rand() is used to generate the 0–1 value, the individual initialization method can be expressed as follows:

(6) $$\;x_{iw}=\{\begin{array}{c} 1,rand>\varepsilon \\ 0,rand\leq \varepsilon \end{array},w=1,\;2,\cdots ,D,$$

where i=1, 2, 3,⋯,SN, ε is a pre-set threshold, which is also a key parameter for individual initialization diversity.

On the other hand, according to the parameters set above, the coverage density of sensors installed on the bridge can be expressed as:

(7) $$\;\rho =\frac{m}{D}\times 100\text{\%},$$

where D is the node number of the bridge FEA model, it is a fixed value for a certain bridge. However, m is variable and its different values correspond to different sensor coverage densities ρ. Dynamically combining ρ to initialize the binary elements of the food source, the improved individual initialization formula can be expressed as follows:

(8) $$\;x_{iw}=\{\begin{array}{c} 1,rand>1-\rho \\ 0,rand\leq 1-\rho \end{array},w=1,2,\cdots ,D.$$

**Figure 2:**
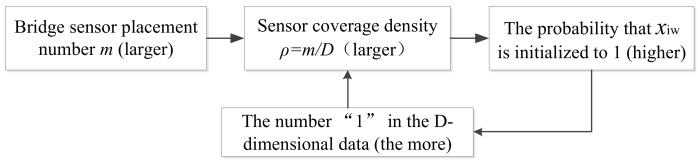
Features of DRCC Initialization.

Thus, the larger the number m of bridge sensor placement, the larger the sensor coverage ρ, and the higher the probability that the corresponding w dimension is initialized to “1” (arranged sensors). This situation just corresponds to the sensor coverage intensity. Therefore, the dispersion degree of individual initialization is dynamically constrained by sensor coverage density ρ of the overall bridge. The corresponding relationship of the above initialization process can be shown in Figure 2.

In the process of using the DRCC initialization, if the number of “1” is less than m, then the initialization is continued with DRCC at the remaining “0” position until the number of “1” in the D-dimensional data equals to m. For the convenience of the program, the elements of the individual are initialized to “0” first, and then they are initialized according to the DRCC step by step.

Through the above modified coding method, we can improve the consistency between the initialization result of food source and the actual demand of bridge sensor placement. Meanwhile, it can maintain the initial dispersion of food sources to the greatest extent, and increase the diversity, so as to improve the global search ability of the algorithm solution process.

### 4.2. Food Source Evolutionary Mechanism Based on MPS

For the convenience of description in the food source evolution stage, it is assumed that the food source $x_{i}$ to be evolved and the randomly selected neighborhood food source $x_{nei}$ are coded as follows (the codes are used only as an example of the MPS process):


$x_{i}$
001000110…1
$x_{nei}$
010010100…1

The two sets of individual code correspond to two potential solutions, each one containing D-dimensional binary data elements. From the probability theory, comparing the elements in the same dimension, if they are the same, the trust value with or without sensors installation in this dimension node is larger. For nodes with larger trust values, they are considered to be relatively good and can be preserved in evolution. Otherwise, if the trust values of some elements are relatively smaller, we can try to randomly select one of them and perform a negation of its values to complete the evolution work.

In order to obtain the trust values of the elements in the selected individual, $x_{i}$ and $x_{nei}$ are matched to get the difference set V:

(9) $$V=\left|x_{i}-x_{nei}\right|\to \{\begin{array}{c} V_{sam}\\ V_{dif} \end{array},$$

where $V_{sam}$ and $V_{dif}$ are sets which separately reflect the dimensions of elements with larger and smaller trust value. When an element of one dimension in the set V is “0”, it indicates that the two selected individuals $x_{i}$ and $x_{nei}$ have the same value in this dimension and its trust value is considered to be large. Naturally, this dimensional information is stored in $V_{sam}$. On the other hand, while an element of another dimension in the set V is “1”, it means that the two selected individuals have different values in this dimension. Then the trust value is considered to be small, and the dimensional information is stored in $V_{dif}$. Obviously, we can know that $V_{sam}\cup^{\text{​}}V_{dif}=\left\{1,\;2,\cdots ,D\right\}$. For the above $x_{i}$ and $x_{nei}$ coding schemes, the following results are satisfied: $V_{sam}=\left\{1,\;4,\;6,\;7,\;9\cdots ,D\right\}$, $V_{dif}=\left\{2,\;3,\;5,\;8,\cdots \right\}$.

**Figure 3:**
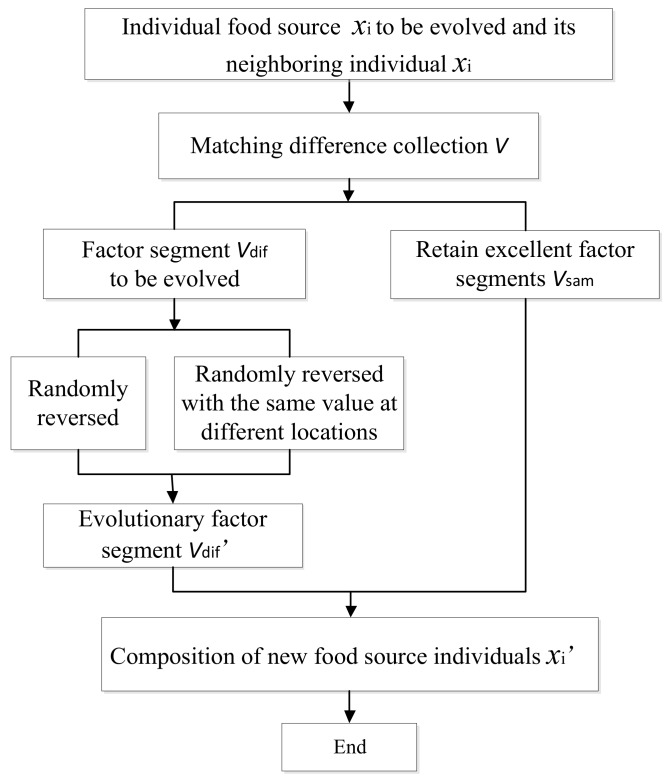
Food source evolution process based on MPS.

The evolutionary idea of the MPS is that after the individual matching of the two food sources, the codes with larger trust value are left behind and a code $v_{di}$ with low trust value is selected in the food source to be evolved, then $v_{di}$ is modified with 0–1 value inversion. At the same time randomly select another element with the same value of $\sim v_{di}$ (this $\sim v_{di}$ is the inverse value of $v_{di}$) in $V_{dif}$ with 0–1 value inversion again to ensure that the number of “1” in the new individual $x_{i}^{'}$ is always equal to the number of sensors to be placed. Of course, if the number of $V_{dif}$ is not enough to perform the second inversion, it is correct to randomly select the same value of $\sim v_{di}$ in $V_{sam}$ to do the same operation to ensure the total sensors number. On the whole, food source evolution process based on MPS is shown in Figure 3.

After the food source individual evolution is completed, the objective function values $fit\left(x_{i}^{'}\right)$ and $fit\left(x_{i}\right)$ are compared. If $fit\left(x_{i}^{'}\right)<fit\left(x_{i}\right)$, then the original food source is replaced by the new one, and the number of stagnation of the new food source is reset to 0. Otherwise, the original food source is retained, and its number of stagnation is increased by 1.

**Figure 4:**
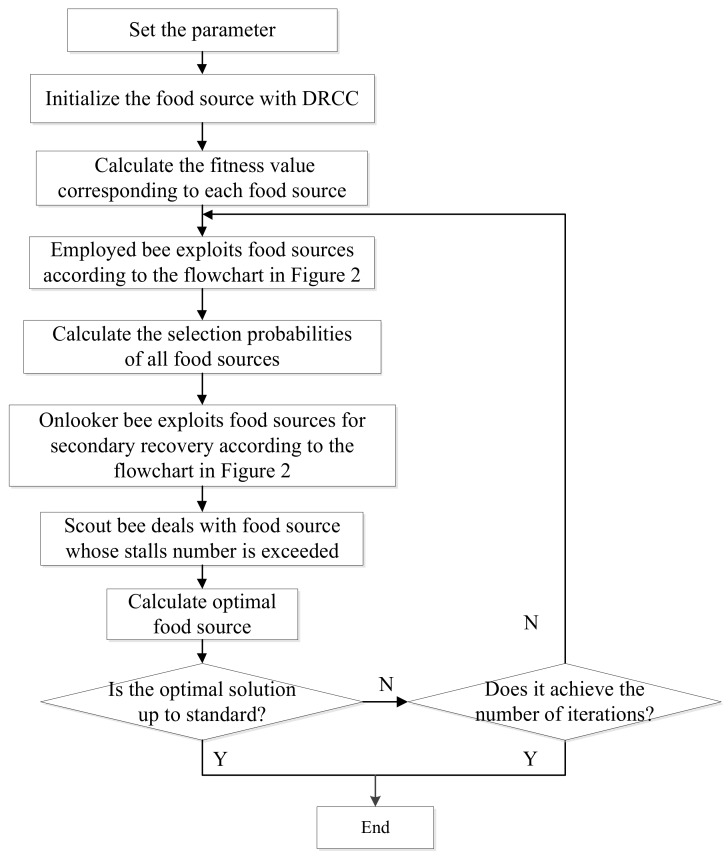
The flowchart of the IABC algorithm.

In the evolutionary with MPS among local individuals, sometimes the local superiority factor cannot significantly improve the current evolutionary results. Due to the diversity of individuals, most food sources can jump out of the limitations of local superior factors after many evolutions, and there are enough new individuals with diversity in the evolution of the whole food source. Moreover, the new individual retains the superior factors of the previous food source and tries not to waste any opportunity to exploit, so that the objective function value of the new food source will tend to be better after evolution. In summary, the flowchart of the IABC algorithm is shown in Figure 4.

The IABC algorithm proposed in this paper is designed for the optimal placement of bridge sensors and the improved strategy combines the actual problems and requirements of the optimal sensor placement closely. We test the algorithm directly through bridge engineering examples in the following section.

## 5. Engineering Example Analysis

### 5.1. Introduction of the Engineering Examples

**Figure 5:**
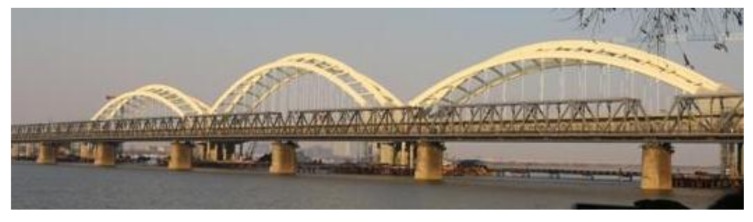
The view of H-Q RB.

The modal data of the Ha-Qi long span railway bridge (abbreviated as H-Q RB) is taken as an example. The bridge is a four-line continuous beam combination one, located in Harbin, China. It was built in 2010 and opened to traffic in 2015. The main bridge length is 624.4 (77 + 3 × 156.8 + 77) meters, and the main span bridge is 156.8 m. The actual scene of the bridge is shown in Figure 5.

**Figure 6:**
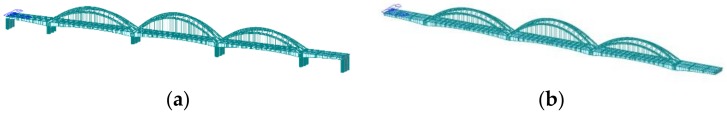
H-Q RB. (**a**) full bridge finite element model; (**b**) the upper structure finite element model.

Using Midas Civil2017 software to establish a FEA to analyze the component with truss unit, there are 1321 nodes and 1393 cells in the model. When optimizing the position of bridge sensor nodes, considering that the low pier has little effect on the overall structure of the bridge, only the upper structure is extracted for calculation. In this way, there are 1251 nodes and 1373 cells. The three-dimensional FEA of the H-Q RB is shown in Figure 6.

For the modal analysis of the upper structure, it is considered that the low-order modes of the structure have larger mode participation coefficients, the first ten order mode shapes data are extracted and various frequencies are shown in Table 1.

Since each node of the bridge FEA has 6 DOF, analyzing the participatory quality results of vibration type and select the Uy direction mode shape vector as the basic data to form a modal matrix $\emptyset_{1251\times 10}$ (the upper structure of the H-Q RB has 1251 nodes). Now, the problem is transformed to select m rows data in $\emptyset_{1251\times 10}$ so that the corresponding maximum non-diagonal element of MAC matrix (objective function value) is minimized. In contrast to practical problems, it means that m nodes are selected as the final sensor placement points in 1251 UyDOF nodes to achieve the best effect of bridge structure health monitoring.

### 5.2. The IABC Algorithm Optimizing the Bridge Sensors Placement

Because the ABC algorithm has strong randomness for group initialization, we try to eliminate the interference of randomness through multiple iterations and set the *m*axCycle to 500. The parameters of the algorithm are shown in Table 2.

In the process of optimizing the placement of the bridge sensors, the first thing to do is to determine the optimal number m of sensors, and then to determine the optimal location of the sensors based on the value of m.

#### 5.2.1. Determining the Optimal Number m of Sensors

Due to the large number of UyDOF nodes in the bridge structure, we search for the value of m which ranges from 1 to 1251 by big step firstly and then lock it by small step in order to quickly find the optimal sensors number. In the process, two kinds of working conditions are compared and analyzed in MATLAB 2017a:

Conditions 1: Using the basic ABC algorithm to search for sensors number of H-Q RB.
Conditions 2: Using the IABC algorithm introducing DRCC initialization and MPS evolution mechanism to search for sensors number of H-Q RB.

(1) Big step search for m value

**Figure 7:**
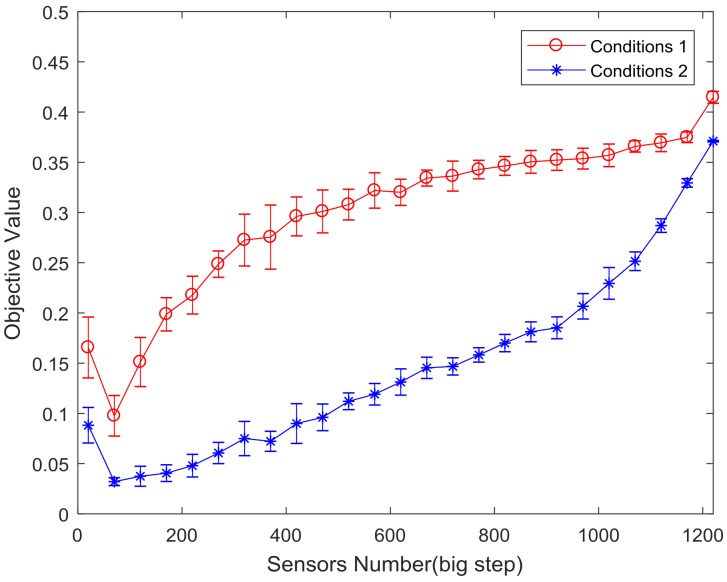
Average curve of the objective function value of Conditions 1 & 2.

Since the number of bridge nodes is as large as 1251, only a few sensors are not sufficient for effective monitoring of the entire bridge according to experience. Here, the value of m is searched from 20 to 1251 in 50 steps. Furthermore, due to the randomness of the colony initialization, a single calculation is not sufficient to determine the optimal sensors number. We adopt the repeated calculation of a single m value for 10 times to get the average value of the objective function, and its curve (containing the error bar) with the change of sensors number is shown in Figure 7.

Comparing the results of Conditions 1 & 2 in Figure 7, both of them lock the approximate range of m near 70, but the results of the optimal objective function calculated under the two conditions are quite different. The optimal objective function values and error results calculated under the two conditions are shown in Table 3.

The data in Table 3 shows that the value of the optimal objective function calculated in Conditions 2 is more than 60% lower than that of Conditions 1, and the stability of the second calculation is raised by 80%. It indicates that the Conditions 2 has a better result in the process of calculating sensors number m. Therefore, only the average of Conditions 2 is used for analysis in the process of searching for m with the small and micro step.

(2) Small step search to determine the value of m

**Figure 8:**
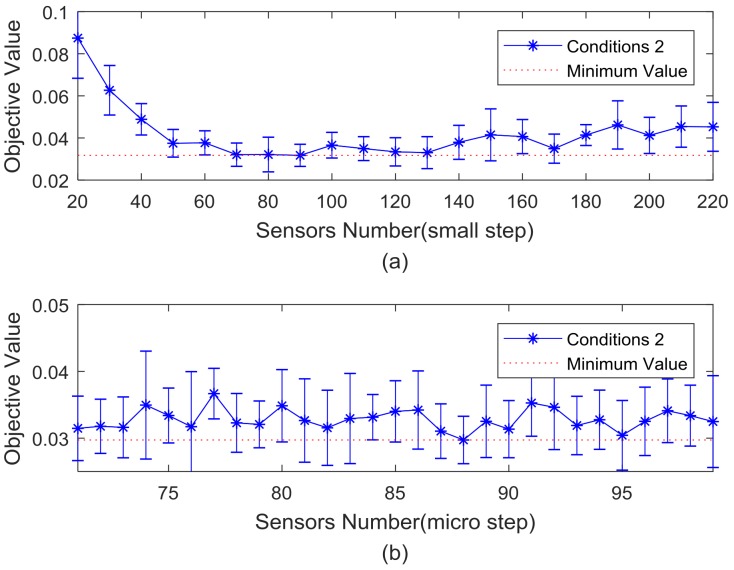
The curve of objective function value in Conditions 2. (**a**) sensors number between 20 and 220; and(**b**) sensors number between 71 and 99.

After a big step searching for the value of m, it can be seen from Figure 7 that the optimal sensors number is between 20 and 220. Furthermore, the best m value is searched in small and micro step. The curve of the average change of objective function value corresponding to the sensors number from 20 to 220 is given in Figure 8a. Based on the results of Figure 8a,b shows the variation of the sensors number from 71 to 99 taking 1 as a step.

Combined with the average value, error minimization of the search results in Figure 8 and the economical consideration, the number of sensor optimal placement of this bridge is determined to 88.

#### 5.2.2. Determining the Optimal Placement of the Sensors

The optimal placement location of the bridge sensors is further solved. In order to analyze the performance of the two improved strategies presented in this paper, the following 4 conditions are studied:

Conditions 1: In the case that the value of m has been determined, the basic ABC algorithm is used to optimize the sensor placement of H-Q RB.
Conditions 2: In the case that the value of m has been determined, the basic ABC algorithm only with the DRCC initialization strategy is used to optimize the sensor placement of H-Q RB.
Conditions 3: In the case that the value of m has been determined, the basic ABC algorithm merely with MPS evolutionary method is used to optimize placement of sensors of H-Q RB.
Conditions 4: In the case that the value of m has been determined, the IABC algorithm which contains DRCC initialization and MPS evolution mechanism is used to optimize the placement of sensors of H-Q RB.

**Figure 9:**
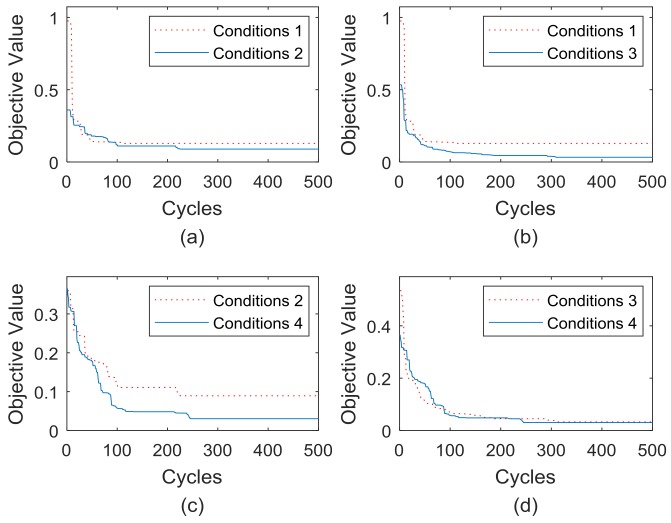
Horizontal comparison of the 4 conditions; (**a**) Comparison of Conditions 1 & 2; (**b**) That of Conditions 1 & 3; (**c**) That of Conditions 2 & 4; (**d**) That of Conditions 3 & 4.

The setting parameters of Table 2 (m=88) is still used, Figure 9 shows a comprehensive comparison of the convergence rate and solving precision under the four conditions.

When using the above four conditions to solve the optimal placement of bridge sensors, the following conclusions can be drawn from Figure 9. Figure 9a shows that the ABC algorithm (only introducing DRCC) has a better convergence rate than the basic ABC algorithm, and the convergence results are also slightly improved.

Figure 9b shows that when the ABC algorithm only introduces the MPS evolution, the convergence rate of the algorithm is significantly improved, and the solution of the objective function value tends to be better.

Figure 9c shows that the IABC algorithm is more capable than the ABC algorithm (only introducing DRCC).

Figure 9d shows that the IABC algorithm is slightly better than the ABC algorithm (only introducing MPS).

On the other hand, the convergence results of the objective function values under the 4 conditions are shown in Table 4, and the percentage improvements of the results under every condition are displayed in Table 5.

The data in Table 5 shows that the basic ABC algorithm merging each improvement strategy proposed in this paper has improved the solution quality of the bridge sensor’s optimal placement, and the MPS evolution mechanism contributes more to the overall performance of the IABC algorithm.

To sum up, for the ABC algorithm integrated with DRCC initialization, such as Conditions 2 and 4 in the early stage of solution, the convergence rate is significantly higher than Conditions 1 and 3. The reason is that DRCC is used to initialize the individual, which is more consistent with the density of the actual bridge sensor coverage, and the individual diversity after initialization is greater, so that the distribution of food sources in the search space is more uniform. As a result, the quality of the entire group solution has been generally improved, which leads to improvements in search speed.

At the later stage of the solving, it can be seen that the optimization accuracy of Conditions 3 and 4 is obviously higher than that of Conditions 1 and 2. The reason is that in the process of food source evolution, the MPS evolution mechanism is adopted to enable the employment bees and onlooker bees to retain the relatively high trust point factor in the exploitation of food sources, which reduces the blindness of search evolution, so that the efficiency of convergence and the accuracy of the algorithm are greatly improved.

#### 5.2.3. Performance Analysis of IABC Algorithm

**Figure 10:**
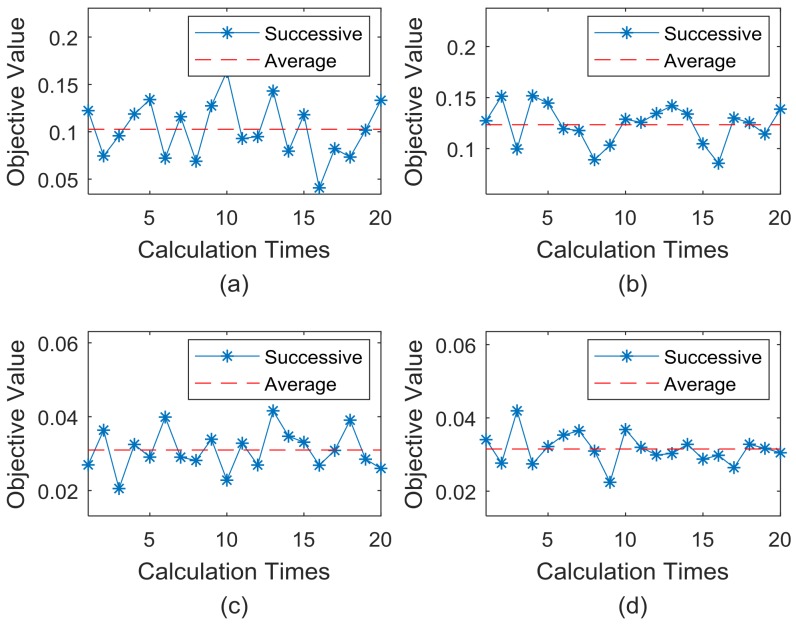
The optimal results after calculating 20 times of the 4 conditions; (**a**–**d**) correspond to the working Conditions of 1, 2, 3 and 4.

In order to analyze the stability of the IABC algorithm’s solution, Figure 10 shows the calculation results of the corresponding optimal objective function value after calculating 20 times for each working condition.

Table 6 shows the average and variance analysis of the results calculated 20 times for each working condition. Table 7 displays the percentage increased in stability for the obtained results of the 4 conditions.

Figure 10 and the data in above two tables show that the basic ABC algorithm merging each improvement strategy proposed in this paper has a better stability than that of the ABC algorithm. And Conditions 4 has the best solution stability, which is 86.23% higher than Conditions 1.

#### 5.2.4. Sensor Placement Results of H-Q RB

Finally, based on the IABC algorithm proposed in this paper, the node number of the sensor placement and its structure description are shown in Table 8. More intuitively, the specific sensor placement in FEA model of the bridge is shown in Figure 10 (the solid dot indicates the location of the sensor installation).

**Figure 11:**
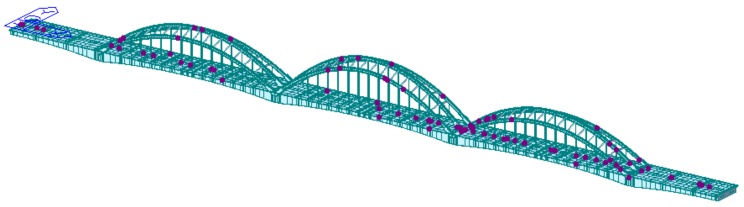
The specific sensor placement in FEA model of H-Q RB.

The 88 sensor placement nodes shown in Table 8 and Figure 11 basically cover the key locations of the H-Q RB, such as main span, side span, sub-midspan bearings, mid-span position, main pier bearings, side arch ribs, suspender, etc., which are more in line with the sensitive point and economic selection principle for bridge health monitoring.

## 6. Conclusions

Aiming at the practical problems existing in the optimal placement of bridge sensors, this paper proposes an IABC algorithm with the DRCC and MPS evolution mechanism based on the ABC algorithm. Then the IABC algorithm is tested by using the FEA model modal data of the H-Q RB. According to the experimental data of the example, the IABC algorithm proposed in this paper has a faster convergence rate and a better solution accuracy. The specific conclusions are as follows:

(1) The introduction of a DRCC method has improved the individual initialization process, making the food source initialization results more in line with the distribution density and actual demand of the bridge sensor arrangement, and increasing the diversity of potential solutions. The validation of engineering examples shows that the improved algorithm can increase the evenness of the distribution of the food source in the search space, so that the quality of the entire colony solution is generally improved and the search speed of the optimal solution is accelerated.
(2) When the employed bees and onlooker bees extract food sources, the evolution mechanism of MPS is introduced to reduce the blindness of search evolution. Engineering examples show that the IABC algorithm has greatly improved the convergence efficiency and the solution accuracy.
(3) The IABC algorithm shows a strong search ability for solving large-scale array data in the process of solving the bridge sensor optimization through the engineering example of H-Q RB and its search speed and solution accuracy are greatly improved compared with the basic ABC algorithm.

## Figures and Tables

**Table 1:** The first ten order modal frequency of upper structure of H-Q RB.

Modal No.	Frequency		Cycle
	(rad/s)	(cycle/s)	(s)
1	1.683166	0.267884	3.732956
2	1.906926	0.303497	3.294929
3	1.926835	0.306665	3.260884
4	2.498795	0.397696	2.514487
5	4.009812	0.638181	1.566953
6	4.706711	0.749096	1.334942
7	5.347616	0.851099	1.174951
8	5.394406	0.858546	1.164759
9	7.161397	1.139772	0.877369
10	9.235729	1.469912	0.680313

**Table 2:** The parameters of the IABC algorithm.

Parameter Name	Value	Parameters	Value
UyDOF Degree of Freedom (D)	1251	Number of iterations (maxCycle)	500
Colony Scale (NP)	20	Upper bound of individual elements (Ub)	1
Food sources Number (FoodNum)	10	Lower bound of individual elements (Lb)	0
Stagnation Number (Trail)	20	Sensor coverage density (ρ)	with the change of m

**Table 3:** Comparison of the calculated results under two conditions.

/	Optimal Objective Function Value	
	Average of 10 Results	Corresponding Error
Condition 1	0.09763	0.02017
Condition 2	0.03207	0.00395
Percentage improved	64.42%	80.42%

**Table 4:** The convergence results of the 4 conditions.

Conditions	Convergence Value
1	0.12875
2	0.089253
3	0.032744
4	0.030323

**Table 5:** The increased percentages under every condition.

Conditions	1	2	3	4
1	0	/	/	/
2	30.68%	0	/	/
3	74.57%	63.31%	0	/
4	76.45%	66.03%	7.39%	0

**Table 6:** Statistics of the results after calculating 20 times.

Conditions	1	2	3	4
Average	0.1026	0.1234	0.0310	0.0315
Variance	0.0305	0.0192	0.0056	0.0042

**Table 7:** The percentage increased in horizontal contrast of 4 conditions.

Conditions	1	2	3	4
1	0	/	/	/
2	37.05%	0	/	/
3	81.64%	70.83%	0	/
4	86.23%	78.13%	25.00%	0

**Table 8:** Sensor placement results (“Lo” represents node location of the sensor placement and “Str” represents the corresponding structure description).

No.	Lo	Str	No.	Lo	Str	No.	Lo	Str	No.	Lo	Str
1	2	SSB	23	274	SM	45	401	SS	67	858	MSA
2	4	SS	24	276	SM	46	402	SS	68	878	MSA
3	7	SS	25	280	SM	47	405	SS	69	915	MSA
4	9	SS	26	281	SM	48	441	SSA	70	933	MSA
5	39	SMB	27	287	SM	49	444	SSA	71	934	MSA
6	41	SM	28	291	SM	50	510	SSA	72	940	MSA
7	56	SM	29	295	SM	51	550	SSA	73	982	MSA
8	63	SM	30	300	SM	52	552	SSA	74	1067	SMS
9	83	SM	31	301	SM	53	620	SSA	75	1089	SMS
10	86	SM	32	304	SM	54	621	SSA	76	1105	SMS
11	104	SM	33	321	SCP	55	664	SSA	77	1114	SMS
12	106	SM	34	324	SM	56	683	SSA	78	1155	SMS
13	212	MCP	35	325	SM	57	685	SSA	79	1159	SMS
14	225	MS	36	340	SM	58	690	SSA	80	1171	SMS
15	235	MS	37	343	SM	59	691	SSA	81	1176	MSS
16	244	MS	38	352	SM	60	706	SSA	82	1208	MSS
17	248	MS	39	358	SM	61	745	SSA	83	1220	MSS
18	250	MS	40	365	SM	62	751	SSA	84	1225	MSS
19	258	MS	41	380	SS	63	752	SSA	85	1236	SMS
20	262	MPB	42	388	SS	64	757	SSA	86	1242	SMS
21	267	SM	43	391	SS	65	809	SSA	87	1247	SMS
22	269	SM	44	398	SS	66	819	SSA	88	1251	SMS

The acronyms of structure description in Table 8 are as follows. SSB: Side span bearings; SS: Side span; SMB: Sub-midspan bearings; SM: Sub-midspan; MCP: Main span crossover position; MS: Main span; MPB: Main pier bearings; SCP: Sub-midspan crossover position; SSA: Sub-midspan side arch ribs; MSA: Main span side arch ribs; SMS: Sub-midspan Suspender; MSS: Main span suspender.
